# Comparative transcriptome analysis of two contrasting wolfberry genotypes during fruit development and ripening and characterization of the *LrMYB1* transcription factor that regulates flavonoid biosynthesis

**DOI:** 10.1186/s12864-020-6663-4

**Published:** 2020-04-10

**Authors:** Cuiping Wang, Yan Dong, Lizhen Zhu, Libin Wang, Li Yan, Mengze Wang, Qiang Zhu, Xiongxiong Nan, Yonghua Li, Jian Li

**Affiliations:** 1 State Key Laboratory of Seedling Bioengineering, Ningxia Forestry Institute, Yinchuan, 750004 China; 2 Agricultural Biotechnology Research Center, Ningxia Academy of Agriculture and Forestry Sciences, Yinchuan, 750002 China; 3 National Wheat Improvement Center, Institute of Crop Science, Chinese Academy of Agricultural Sciences, Beijing, 100081 China; 4 Biochip Research Center, the General Hospital of Ningxia Medical University, Yinchuan, 750004 China

**Keywords:** *Lycium barbarum*, *L*. *ruthenicum*, Illumina sequencing, Anthocyanin synthesis, Sugar metabolism, MYB transcription factor

## Abstract

**Background:**

*Lycium barbarum* and *L. ruthenicum* have been used as traditional medicinal plants in China and other Asian counties for centuries. However, the molecular mechanisms underlying fruit development and ripening, as well as the associated production of medicinal and nutritional components, have been little explored in these two species.

**Results:**

A competitive transcriptome analysis was performed to identify the regulators and pathways involved in the fruit ripening of red wolfberry (*L. barbarum*) and black wolfberry (*L. ruthenicum*) using an Illumina sequencing platform. In total, 155,606 genes and 194,385 genes were detected in red wolfberry (RF) and black wolfberry (BF), respectively. Of them, 20,335, 24,469, and 21,056 genes were differentially expressed at three different developmental stages in BF and RF. Functional categorization of the differentially expressed genes revealed that phenylpropanoid biosynthesis, flavonoid biosynthesis, anthocyanin biosynthesis, and sugar metabolism were the most differentially regulated processes during fruit development and ripening in the RF and BF. Furthermore, we also identified 38 MYB transcription factor-encoding genes that were differentially expressed during black wolfberry fruit development. Overexpression of *LrMYB1* resulted in the activation of structural genes for flavonoid biosynthesis and led to an increase in flavonoid content, suggesting that the candidate genes identified in this RNA-seq analysis are credible and might offer important utility.

**Conclusion:**

This study provides novel insights into the molecular mechanism of *Lycium* fruit development and ripening and will be of value to novel gene discovery and functional genomic studies.

## Background

*Lycium barbarum* and *L. ruthenicum* belongs to the *Lycium* genus of the Solanaceae family; these species are widely distributed in the arid and semiarid areas of northwestern China and have been extensively used as traditional medicine plants in China for thousands of years [1]. The fruit of *L. barbarum and L. ruthenicum* are very important agricultural and biological products, with advantages of having both medicinal and nutritional functions. For instance, the fruit can be used for enhancing eyesight, curing heart disease and improving abnormal menstruation [2]. In recent years, modern pharmacological studies have begun to investigate the biochemical mechanisms of the medicinal effects of these two *Lycium* species and found that the health-promoting characteristics were primarily attributable to the production and accumulation of bioactive compounds [3]. The red fruit of *L. barbarum* contain mainly polysaccharides, flavonoids and carotenoids [4, 5], while the major phytochemicals in the black fruit of *L. ruthenicum* are anthocyanins, essential oils and polysaccharides [2, 6, 7].

Traditionally, *Lycium* breeding efforts have concentrated on various agronomic traits, such as yield and the ability to withstand biotic and abiotic stresses. However, with increasing consumer interest in health protection, the breeding of *Lycium* may gradually shift to nutritional and health-protective varieties in the near future [8]. As a result, more comprehensive knowledge of genes encoding enzymes of secondary metabolism and regulatory genes is necessary to breed varieties that have increased benefits. Researchers have previously studied *Lycium* species through various ways, including simple sequence repeat (SSR) mining and validation, genetic population construction, and genetic diversity analysis [9]. However, there are few genomic resources for *Lycium*. To date, no genomic sequence data of the *Lycium* genus have been reported. Gene sequences are usually obtained from comparisons between other species of Solanaceae [10].

Fruit ripening is a genetically programmed, highly coordinated, and irreversible process that relies on a chain of physiological, biochemical and organoleptic changes that eventually result in the development of a mature and edible fruit [11–13]. Fruit development and ripening have a substantial influence on the levels of various bioactive compounds, such as flavonoids and polyphenolics, and ultimately affect the quality of the fruit [14]. The underlying mechanisms of fruit development and ripening have been extensively studied in tomato but are not well explored in *Lycium*. Shinozaki et al. (2018) presented a global analysis of the tomato fruit transcriptome through tissues, cell types, development, and fruit topography, and revealed complex programs that were regulated in coordination across cell/tissue types and developmental stages [15]. Flavonoids and sugars, with functions in pigmentation, fertility and signaling for the former and taste for the latter, are two kinds of important components in *Lycium*. These two active substances undergo important changes during fruit development, with great differences between *L. barbarum* and *L. ruthenicum*. Anthocyanins, a major group of flavonoids, increase steadily during fruit development of *L. ruthenicum* and reach maximum levels at the last stage, but they are not detected at all stages in *L. barbarum* fruit [16]. The content and composition of sugars not only determine the basic material supply in fruit during wolfberry fruit quality development but also affect substrates involved in many secondary metabolites and active substance synthesis [17]. For instance, the contents of fructose and glucose in wolfberry fruit increase with fruit growth and development, but the content of sucrose decreases [18]. However, no reports have examined the sugar content of *L. ruthenium*. Fortunately, genomic studies that catalog the full genetic repertoire can offer clues to complex regulatory networks and help us identify genes involved in the metabolism of bioactive compounds [19]. As important bioactive compounds in the fruit of *L. barbarum*, flavonoids have been extensively studied; Chen et al. (2017) identified genes in the flavonoid biosynthesis pathway of *L. barbarum* by transcriptome analysis [20]. However, the mechanisms controlling the species differences in flavonoid biosynthesis between *L. barbarum* and *L. ruthenicum* remain unknown.

The aim of this study was to comparatively analyze the transcriptomes of two contrasting *Lycium* genotypes, red fruit and black fruit wolfberry (*L. barbarum* and *L. ruthenicum*, respectively), during the ripening period to identify genes associated with the biosynthesis of bioactive compounds. We also sought to identify key potential regulators of secondary metabolite biosynthesis involved in the development and ripening of wolfberry fruit. A promising candidate flavonoid regulating transcription factor, *LrMYB1*, was characteristic of transgenic *L. barbarum.* This study offers an important genetic resource for revealing the genes associated with development and ripening and provides further insights into the identification of key potential pathways and regulators involved in the development and ripening of *Lycium.* Eventually, the information here may provide basic information for the molecular breeding of *Lycium* varieties.

## Results

### Sequencing and transcript assembly of identified genes expressed during fruit ripening

A total of 18 cDNA libraries prepared from fruit flesh samples at the three critical ripening stages (with three biological replicates for each stage and each *Lycium* species) were constructed. The raw sequencing data were checked for quality and subjected to data filtering. In total, 49,100,240~53,878,068 and 43,848,978~51,056,242 raw reads were generated from RF and BF, respectively. After removing low quality short sequences, 41,997,634~49,545,044 and 46,722,298~52,399,006 clean reads were obtained for RF and BF, respectively. All clean reads were deposited in the NCBI Short Read Archive (SRA) database under accession number PRJNA483521. A summary of the sequencing data is listed in Table 1. The contigs were assembled into 155,606 unigenes for RF with a mean length of 1287 bp and an N50 of 1939 bp and 194,385 unigenes for BF with a mean length of 1223 bp and an N50 of 1835 bp (Table 2, Additional file 1).

**Table 1:** Overview of the RNA-seq data from RF and BF at each of the three fruit developmental stages

Sample	Name	Raw Reads	Clean reads	Clean bases	Error (%)	Q20 (%)	Q30 (%)	GC (%)
LbG1	RFS1	46132734	44796750	6.72G	0.02	97.15	92.65	42.97
LbG2	RFS1	47970842	4656150	6.98G	0.02	97.31	92.99	42.86
LbG3	RFS1	45495590	44165234	6.62G	0.02	97.32	92.98	42.91
LbT1	RFS3	51056242	49545044	7.43G	0.02	97.41	93.13	42.26
LbT2	RFS3	50074818	48300496	7.25G	0.02	96.71	91.72	42.06
LbT3	RFS3	48539438	46358492	6.95G	0.02	96.99	92.37	42.06
LbR1	RFS2	48230562	46211844	6.93G	0.02	96.78	91.97	41.59
LbR2	RFS2	47494602	46194950	6.93G	0.02	96.15	90.78	40.49
LbR3	RFS2	43848978	41997634	6.3G	0.02	96.87	92.22	41.31
LrG1	BFS1	53878068	52399006	7.86G	0.02	97.37	93.11	42.71
LrG2	BFS1	50815816	49325838	7.4G	0.02	97.22	92.78	42.99
LrG3	BFS1	49535828	48152026	7.22G	0.02	97.37	93.10	42.90
LrT1	BFS3	53206854	51476830	7.72G	0.02	96.73	91.71	42.42
LrT2	BFS3	49561342	48105208	7.22G	0.02	97.33	93.01	42.49
LrT3	BFS3	49100240	46722298	7.01G	0.02	96.84	92.08	42.35
LrR1	BFS2	50534672	48440070	7.27G	0.02	96.93	92.27	41.83
LrR2	BFS2	49687764	47622698	7.14G	0.02	96.96	92.30	42.01
LrR3	BFS2	51493156	49420204	7.41G	0.02	97.00	92.41	41.92

*BF* black fruit of *L. ruthenicum*, *RF* red fruit of *L. barbarum*; S1: 10 DAF; S2: 25 DAF; S3: 40 DAF. *DAF* days after flowering

**Table 2:** Characteristics of the assembled transcripts and unigenes

	Min Length	Mean Length	Median Length	Max Length	N50	N90	Total Nucleotides
RF							
Transcripts	201	949	498	15810	1745	348	221962508
Genes	201	1287	899	15810	1939	588	200333158
BF							
Transcripts	201	944	526	16681	1654	363	261681100
Genes	201	1223	844	16681	1835	559	237719269

### Functional annotation by similarity searches

**Fig. 1:**
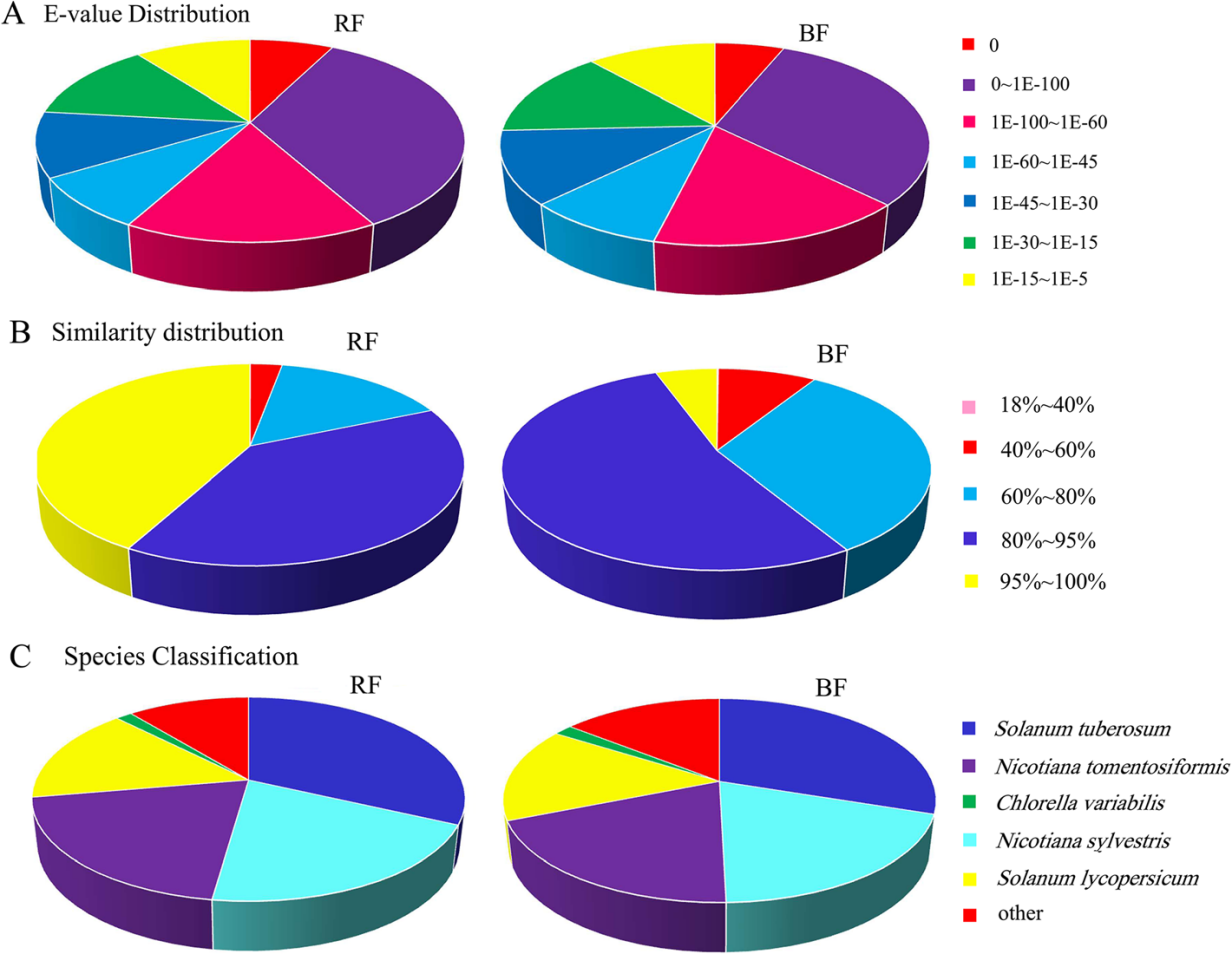
Distribution of the homology searches of unigenes using the nonredundant (NR) protein database. **a** E-value distribution of the top BLASTx hits against the NR database for each unigene. **b** Similarity distribution of the top BLASTx hit against the NR database for each unigene. **c** Species distribution of unigenes matching the top five species using BLASTx in the NR database. RF: red fruit of *L. barbarum*; BF: black fruit of *L. ruthenicum*

These assembled unigenes were functionally annotated by aligning the gene sequences against the NCBI nonredundant protein (NR), Swiss-Prot protein, Clusters of Orthologous groups (COG), Kyoto Encyclopedia of Genes and Genomes (KEGG), Gene Ontology (GO), and Protein family (Pfam) databases using BLASTx, and against the nucleotide database (NT) by BLASTn with an E-value threshold of 1e-5. Using this approach, 72.67% of the total unigenes (155,606) for RF and 71.15% of the total unigenes (138,322) for BF were annotated. The remaining unigenes were predicted by the ESTs. The E-value, identity, and species distribution were analyzed. According to the E-value distribution in the NR databases, 66.4 and 62.7% of the matched unigenes for RF and BF, respectively showed homology (<1e-45) (Fig. 1a). For the similarity distribution of the predicted proteins, 93.2 and 91% of the sequences for RF and BF, respectively, had a similarity higher than 60% (Fig. 1b). The species distribution of the top BLAST hits in the NR database for the *Lycium* fruit transcriptome showed that these sequences had the greatest number of matches with genes from *Solanum tuberosum,* followed by *N. sylvestris*, *N. tomentosiformis*, and *S. lycopersicum* (Fig. 1c).

### Differences in gene expression between RF and BF

**Fig. 2:**
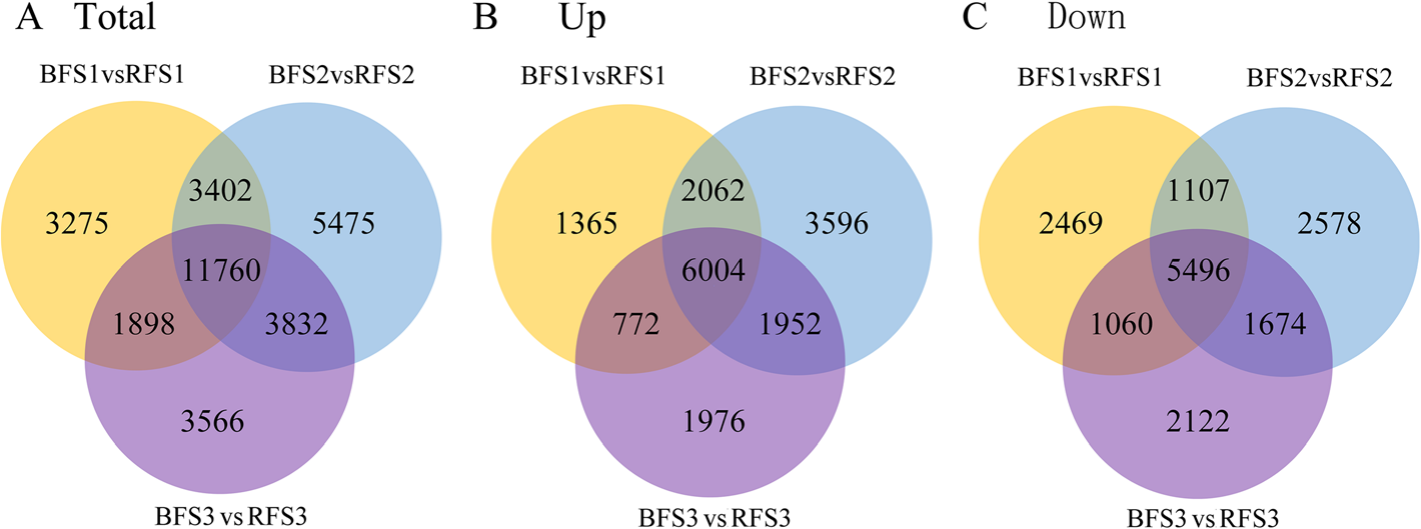
Differential expression analysis of RF and BF at different stages (S1-S3) during fruit development and ripening. **a** Total differential expression of unigenes (DEGs); **b** upregulated DEGs; **c** downregulated DEGs. S1: 10 DAF; S2: 25 DAF; S3: 40 DAF. DAF: days after flowering

In this study, an estimated absolute value of log_2_(−fold change) ≥1 and adjusted *P* < 0.05 were used as thresholds for detecting significant differences in gene expression between two samples (the former was the control and the latter was the treatment group) during fruit development. In total, 20,335 genes were differentially expressed at stage S1 between BF and RF, including 10,203 upregulated genes and 10,132 downregulated genes. At stage S2, 24,469 genes were differentially expressed, with 13,614 upregulated genes and 10,855 downregulated genes. At stage S3, 21,056 genes were differentially expressed, with 10,704 upregulated genes and 10,352 downregulated genes. Among the 13,614 upregulated genes at stage S2, 7956 genes were still upregulated at stage S3, whereas 466 of the upregulated genes had the opposite expression pattern at stage S3 (Fig. 2, Additional file 2).

### Verification of the expression of various DEGs detected during fruit ripening

Gene Ontology (GO) was used to compare the unigenes, which included 63,519 (40.82% of all cleaned unigenes) sequences with the same cut-off E-value as that used for the supplemental and functional annotations (Additional file 3). In total, 113,081 annotated transcripts were identified, representing approximately 72.47% of all the cleaned unigenes. According to the GO analysis, 20,335 DEGs from stage S1 could be divided into three major categories: biological processes, cellular components, and molecular function. Among the clusters of biological processes, cellular processes and metabolic processes were the two largest groups with 2644 DEGs. In the cellular component cluster, 1343 DEGs in cells and cell parts were dominant. In the molecular function group, binding and catalytic activity were the largest two subcategories with 617 DEGs. We found that 24,469 DEGs from stage S2 could be distributed into three main GO categories, including the cluster of biological processes, with 2805 DEGs, molecular function, with 1402 DEGs, and cellular components, with 627 DEGs. In addition, there were 21,056 DEGs from stage S3, including 2674 DEGs in biological processes, 1374 DEGs in molecular function and 622 DEGs in cellular components.

All the annotated unigenes were mapped to the KEGG database to define the cellular pathways containing all the unigenes, which helped us further understand the potential functions of the annotated unigenes at the transcriptomic level. In stage S1, 5184 DEGs were mapped 122 KEGG pathways (Additional file 4), and the 20 top KEGG pathways with the highest representation are shown in Additional file 5. In stage S2, 6658 DEGs were mapped to 122 KEGG pathways (Additional file 4), and the 20 top KEGG pathways with the highest representation are shown in Additional file 5. In stage S3, 5679 DEGs were mapped to KEGG pathways (Additional file 4), and the 20 top KEGG pathways with the highest representation are shown in Additional file 5. Of these KEGG pathways, terpenoid biosynthesis, steroid biosynthesis, glutathione metabolism, phenylalanine metabolism, pentose phosphate pathway, butanoate metabolism, synthesis and degradation of ketone bodies, and cysteine and methionine metabolism were the KEGG pathways identified in all stages (S1, S2, and S3). Flavonoid biosynthesis was identified in both stages S2 and S3, but not in stage S1. Fructose and mannose metabolism were identified in both stages S1 and S2, but not in stage S3.

### DEGs involved in phenylpropanoid biosynthesis, flavonoid biosynthesis, anthocyanin biosynthesis, and sugar metabolism during fruit ripening of RF and BF

Because RFs are rich in polysaccharides (LBP), flavonoids and carotenoids [4, 5], whereas BFs mainly contain anthocyanins, essential oils and polysaccharides, the DEGs involved in flavonoid biosynthesis, anthocyanin biosynthesis, sugar and betaine metabolism during fruit ripening were analyzed in this study. First, phenylalanine is converted to naringenin chalcones through the phenylpropanoid pathway successively catalyzed by phenylalanine ammonia lyase (PAL, 6 DEGs), cinnamate 4-hydroxylase (C4H, 3 DEGs), 4-coumarate CoA ligase (4CL, 7 DEGs) and chalcone synthase (CHS, 3 DEGs). The stereo-specific cyclization product is subsequently converted into naringenins or flavanones by chalcone synthase (CHI, 1 DEG). The hydroxylation of flavanones by flavanone 3-hydroxylase (F3H, 2 DEGs) yields dihydrokaempferols, which are subsequently converted to dihydroquercetin by flavonoid 3′-hydroxylase (F3’H, 1 DEG) or to dihydromyricetin by flavonoid 3′5’-hydroxylase (F3’5’H, 2 DEGs). Last, the dihydrokaempferols, dihydroquercetins and dihydromyricetins are converted to flavonols by flavonol synthase (FLS, 1 DEG). In the anthocyanin branch, dihydroflavonol 4-reductase (DFR, 0 DEG) converts dihydrokaempferol, dihydroquercetin, and dihydromyricetin to leucopelargonidin, leucocyanidin, and leucodelphinidin, respectively. Leucoanthocyanidin dioxygenase (LDOX, 1 DEG), which is also known as anthocyanidin synthase (ANS), catalyzes the oxidation of leucopelargonidin, leucocyanidin, and leucodelphinidin to pelargonidin, cyanidin, and delphinidin, respectively. The final modification steps for the production of colored and stable compounds are the glycosylation of pelargonidin, cyanidin, and delphinidin by UDP-glucose flavonoid 3-O-glucosyl transferase (UFGT). Eventually, only cyanidin-3-glucoside and delphinidin-3-glucoside can be methylated by methyltransferase (MT) and then converted to peonidin-3-glucoside and petunidin- or malvidin-3-glucoside, respectively. The synthesis of PAs branches off the anthocyanin pathway after the reduction of leucoanthocyanin (or anthocyanin) to catechins (or epicatechins) by leucoanthocyanidin reductase (LAR, 1 DEG) and anthocyanidin reductase (ANR, 1 DEG) [21].

Sugars are critical components of *Lycium* fruit. Several of the genes associated with sugar metabolism and cell wall metabolism were identified as being differently expressed between the two *Lycium* species, including genes encoding alpha-galactosidase (5 DEGs), phosphoglucomutase (2 DEGs), beta-fructofuranosidase (6 DEGs), 6-phosphofructokinase 1 (1 DEG), hexokinase (2 DEGs), raffinose synthase (4 DEGs), beta-fructofuranosidase (5 DEGs), sucrose-phosphate synthase (4 DEGs), sucrose synthase (6 DEGs), beta-glucosidase (21 DEGs), fructokinase (4 DEGs), and phosphoglucomutase (2 DEGs).

### Comparative RNA-seq profile of anthocyanin- and sugar-related genes in the fruit of the two *Lycium* species

**Fig. 3:**
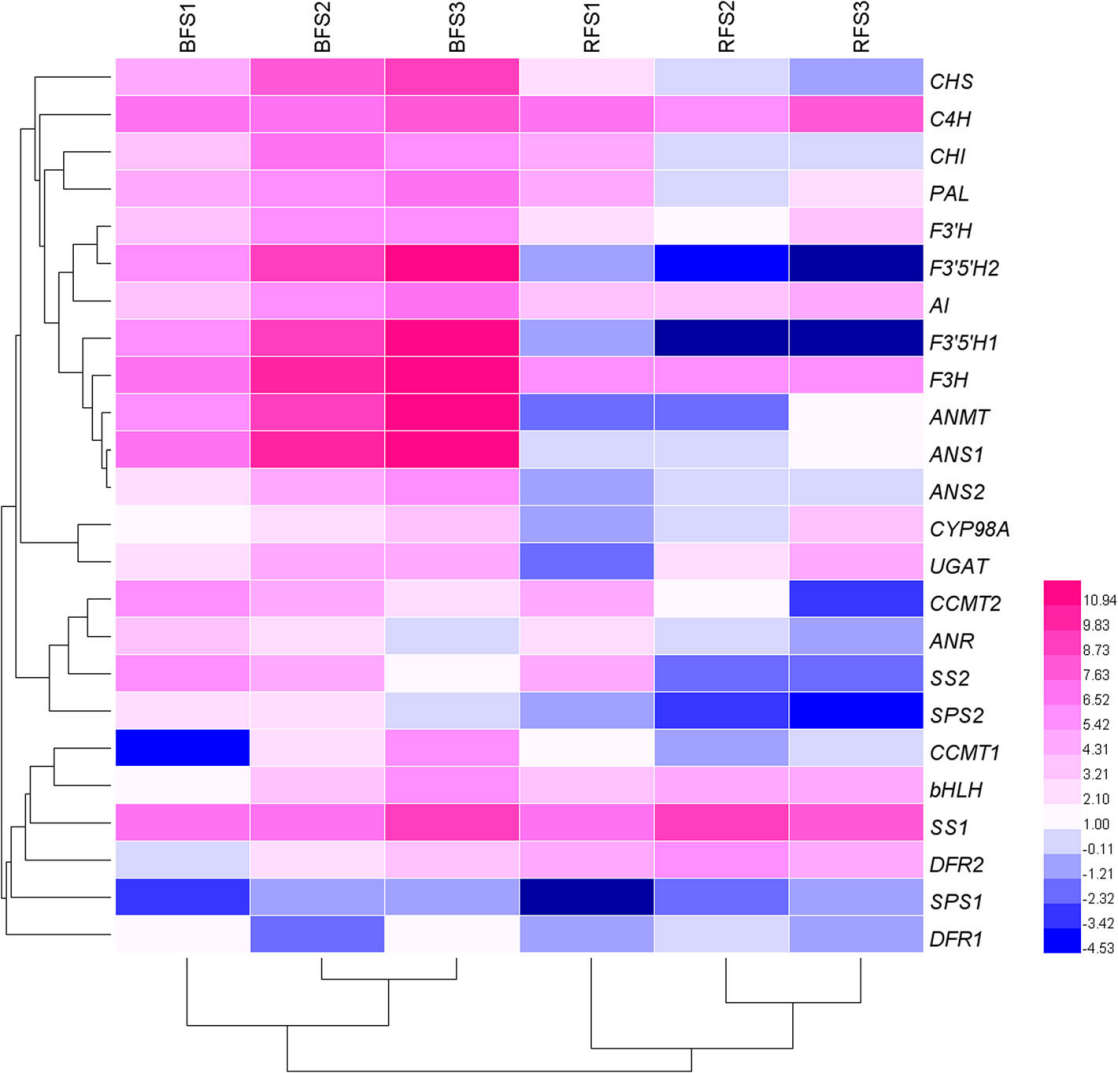
Heat map of the expression levels of DEGs constructed via HemI 1.0 [22]. The DEGs are involved in anthocyanin biosynthesis and sugar metabolism. The clustering of samples on the X-axis and Y-axis is based on the similarity of gene expression patterns. BF: Black fruit of *L. ruthenicum*; RF: red fruit of *L. barbarum*

To comparatively summarize the expression of the anthocyanin and sugar-related genes in both species, RNA-seq data derived from the three stages were profiled. The FPKM values of the anthocyanin- and sugar-related genes in the two *Lycium* fruit are shown in Additional file 6. The FPKM values of the anthocyanin-related genes (*F3H*, *ANS1*, *F3’5’H*, *ANMT* and *ANS1*) were much higher in the BF than in RF. For example, the FPKM of *F3H* reached 4224 in the BF (Fig. 3 and Additional file 7). Moreover, the FPKM values of most anthocyanin-related genes tended to increase trends and reached into the thousands in S3 (Fig. 3, Additional files 6 and 7). Conversely, in all three RF stages, the FPKM value of nearly all the anthocyanin related genes detected was less than 200. These results suggested that the greater amount of anthocyanins in BF than RF correlated with the higher expression of these relevant genes. The FPKM values of sugar-related genes showed different spatial and temporal patterns (Fig. 3 and Additional file 7). The FPKM values of SS2 and SPS2 were higher in the BF than in the RF. However, homologous SS1 and SPS1 showed no significant difference between BF and RF. SS2 and SPS2 decreased, but SS1 and SPS1 increased during fruit development. The FPKM value of AI was higher in the BF than in the RF, and AI showed an increasing trend during fruit development (Fig. 3 and Additional file 7).

**Fig. 4:**
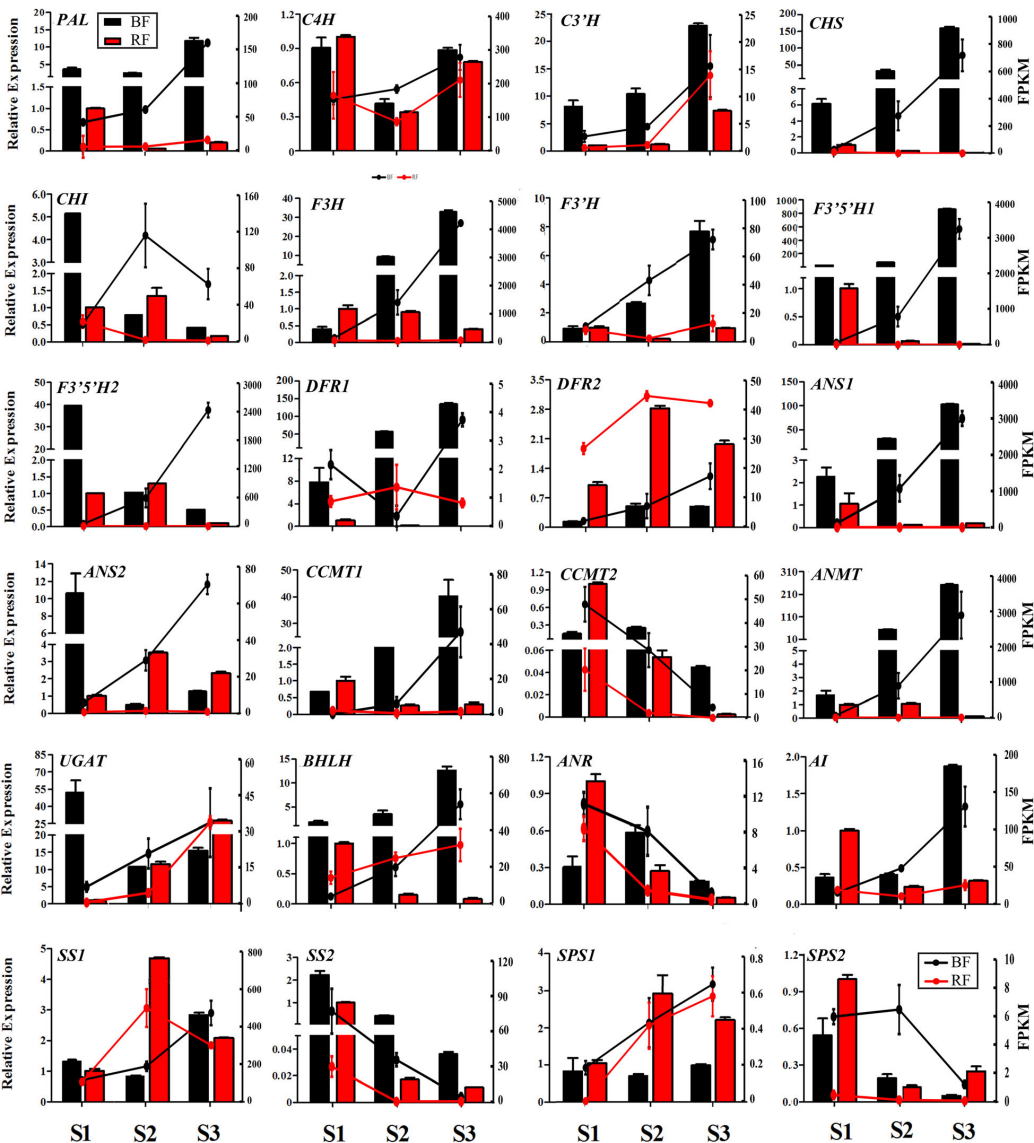
FPKM values calculated from the transcriptomic data, and validation by qRT-PCR of genes involved in anthocyanin biosynthesis and sugar metabolism in ripening RF (*L. barbarum*; red lines and bars) and BF (*L. ruthenicum*; black lines and bars). The data are the means ± SEs of three biological repetitions, *n* = 3

To validate the results of the RNA-seq analysis, quantitative real-time PCR (qRT-PCR) assays were performed for DEGs involved in anthocyanin biosynthesis and sucrose metabolism. As shown in Fig. 4, qRT-PCR was performed to confirm the expression patterns of 24 of the anthocyanin and sugar-related genes during BF and RF ripening (S1-S3). Consistent with the RNA-seq data, the transcripts of ten anthocyanin-related genes (*PAL*, *CHS*, *F3H*, *F3’H*, *F3’5’H*, *DFR1*, *ANS1*, *CCMT1*, *ANMT* and *bHLH*) gradually decreased throughout RF ripening, while those transcripts were consistently expressed in all three BF stages. In particular, the transcript abundance of *F3H*, *F3’5’H1* and *ANMT* increased dramatically (by 82-, 37.4- and 150.1-fold, respectively) from S1 to S3 in BF. The transcript abundance of *C3’H* increased by 8.4-fold from S1 to S3 in the BF, whereas *C3’H* was consistently expressed at low levels during RF ripening. However, the transcripts of *CHI* and *F3’5’H2* gradually decreased throughout BF ripening, while they peaked at S2 but then decreased in the RF. Similarly, the transcripts of *CCMT2* and *ANR* decreased consistently during RF ripening, while they peaked at S2 and decreased thereafter in the BF. Two genes, *UGAT* and *ANS2*, gradually decreased during BF ripening, while they were consistently expressed throughout RF ripening. The *C4H* gene showed a similar expression pattern in both BF and RF, with decreasing expression at stage S2 but increasing expression at stage S3. Specifically, the *DFR2* transcript peaked at S2 and decreased thereafter during RF ripening, while it was constantly expressed at low levels throughout the BF ripening process. Five sugar-related genes were also validated with qRT-PCR. For instance, the transcript abundance of AI increased 5.2-fold from S1 to S3 in the BF, while it gradually decreased during RF ripening. The *SS1* gene transcript consistently increased during BF ripening, peaked at S2 and decreased thereafter in the RF. The *SPS1* transcript peaked at S2 in the RF, and the abundance remained high thereafter. The transcripts of the *SS2* and *SPS2* genes gradually decreased during fruit ripening processes of both *Lycium* species.

### Variation in sugar content during the ripening of BF and RF fruit

**Fig. 5:**
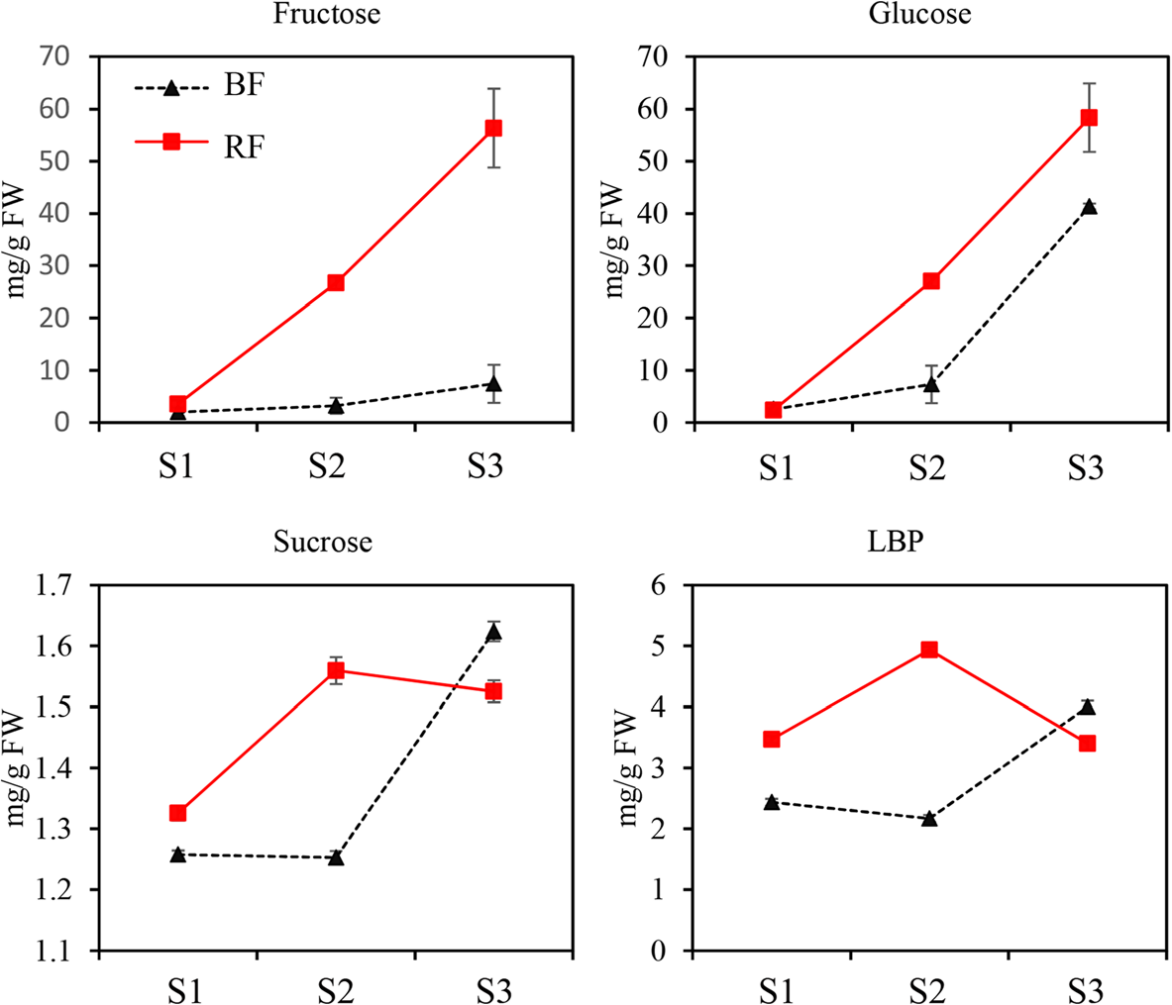
Dynamics of soluble sugar and *Lycium barbarum* polysaccharide (LBP) accumulation during fruit development and ripening. The data are the means ± SEs of three biological repetitions, *n* = 3

Since the expression of the number of genes implicated in sugar metabolism varied greatly during fruit development and ripening, an attempt was made to monitor the dynamics of sugar content. Glucose and fructose accumulated throughout the three stages of fruit development in *L. barbarum* and *L. ruthenicum* and the concentration was higher in the RF than in the BF. The concentrations of sucrose and LBP increased before 25 DAF and then decreased in the RF. However, in the BF, their concentration remained constant, slightly decreased before 25 DAF, and then rapidly increased to a higher level at 40 DAF. Moreover, the contents of sucrose and LBP were higher in the RF than in the BF before 25 DAF but lower in the RF than in the BF at 40 DAF (Fig. 5).

### LrMYB1 encodes an R2R3 domain protein and is located in the nucleus

**Fig. 6:**
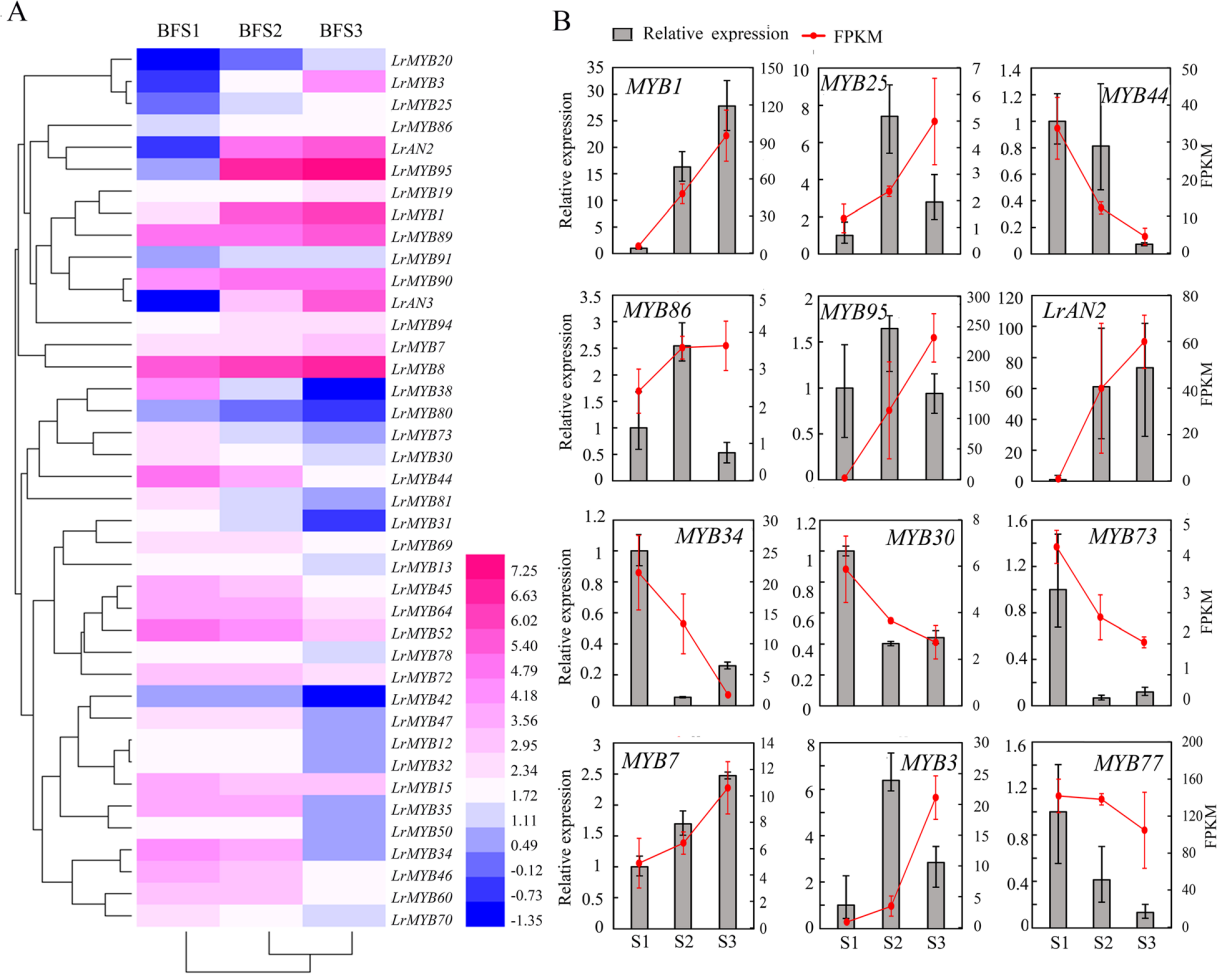
**a** Characterization of differentially expressed MYB transcription factors during fruit development in *L. ruthenicum*. Heat map of the expression levels of DEGs constructed via HemI 1.0 [22]. The clustering of samples on the X-axis and Y-axis is based on the similarity of gene expression patterns. **b** FPKM values calculated from the transcriptomic data, and the verification of partial differentially expressed genes by qRT-PCR. The data are the means ± SEs of three biological repetitions, *n* = 3

We identified 82 MYB TFs (32 R2R3-MYBs, 45 1R-MYBs, 4 3R-MYBs and 1 4R-MYB) in *L. ruthenicum* after completely removing repeated and redundant sequences [19]. To confirm the MYB transcription factors that might play vital roles in the development and maturation of BF, the DEGs in different developmental stages in BF were annotated and classified by annotations in the NR, NT, and Swiss-Prot database and via SMART. Of these 82 MYB TFs, the expression of 15 MYB TFs was upregulated during fruit development and ripening, and that of 26 MYB TFs was downregulated (Fig. 6a, Additional file 8). The expression of partial genes was verified by qRT-PCR and showed a trend similar to that exhibited by the transcriptomic data (Fig. 6)b.

**Fig. 7:**
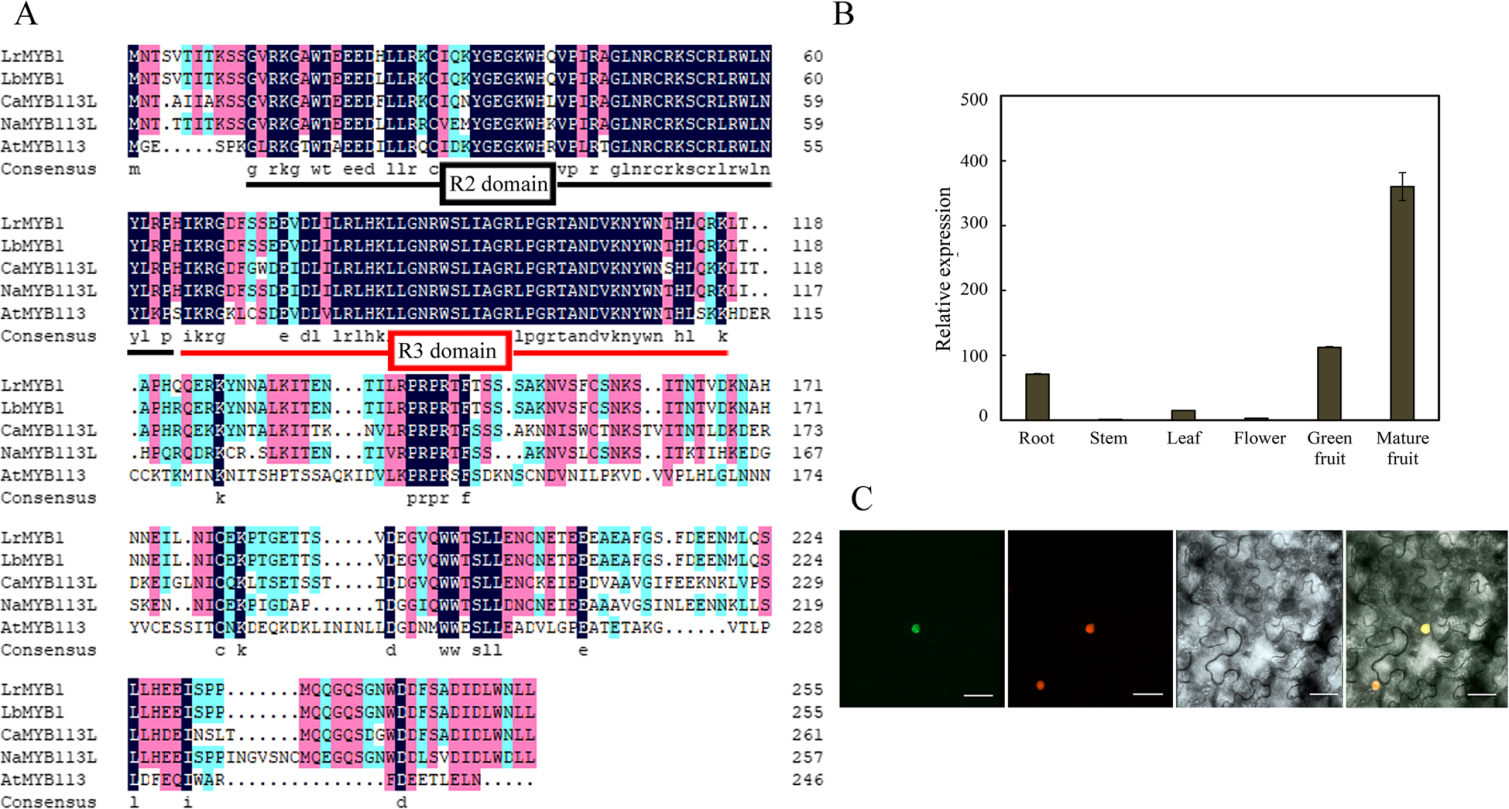
Amino acid, organ-specific expression and subcellular localization analysis of LrMYB1. **a** Comparison of predicted LrMYB1 protein sequences with anthocyanin-related MYB proteins of other species. The R2R3-binding domain is underlined. **b** Quantitative RT-PCR analysis of LrMYB1 expression in the roots, stems, leaves, flowers, green fruit and mature fruit of *L. ruthenicum*. Three independent experiments were performed. The data are the means ± SEs of three biological repetitions, *n* = 3. **c** Colocalization of the 35S:LrMYB1:GFP signal and nucleus in a single leaf epidermal cell. From left to right: merged, 35S:LrMYB1:GFP, DAPI staining, bright field. Bars = 10 μm

Several *Lycium* genes with increased expression during fruit development and ripening are promising candidate genes for flavonoid/anthocyanin synthesis. Based on the sequence alignment, *LrMYB1* had high sequence identity to *CaMYB113L* and *NaMYB113L* among the R2R3 subfamily of plants belonging to the Solanaceae family in the open reading frame at the amino acid level; moreover, *LrMYB1* appears to be a new member of the R2R3 MYB gene family. Additionally, *LrMYB1* had the highest similarity to *AtMYB113* (At1g66370) in *Arabidopsis*, suggesting that it might have functions similar to those of AtMYB113. Consequently, we selected *LrMYB1* to clone for functional characterization. The full-length *LrMYB1* clone (GenBank Accession No. DQ109673) was 1016 bp contained an open reading frame of 771 bp and encoded a putative protein of 256 amino acids with a predicted molecular mass of 29.54 kDa and a pI of 7.66. Further analysis of the deduced amino acid sequence revealed that this protein was a typical R2R3 MYB protein with two typical SANT MYB DNA-binding domains (Fig. 7)a.

The expression analysis of *LrMYB1* via quantitative RT-PCR in mature plants revealed that *LrMYB1* transcripts were more abundant in the roots and fruits than in the stems, leaves and flowers, and were more abundant in mature fruit than in green fruit (Fig. 7)b. We also performed an in silico analysis using Prot Comp 9.0 (http://linux.softberry.com) software to characterize the subcellular localization of LrMYB1, and LrMYB1 was predicted to be a nucleus-localized protein. We then analyzed a transiently transformed *Nicotiana tabacum* plant in which GFP was translationally fused to *LrMYB1* under the control of the 35S promoter. Confocal microscopy revealed that GFP colocalized with DAPI red staining, a nuclear-specific dye, which indicated that LrMYB1 was indeed targeted to the nucleus (Fig. 7)c.

### Constitutive expression of *LrMYB1* in transgenic *L. barbarum* increased the total flavonoid content

**Fig. 8:**
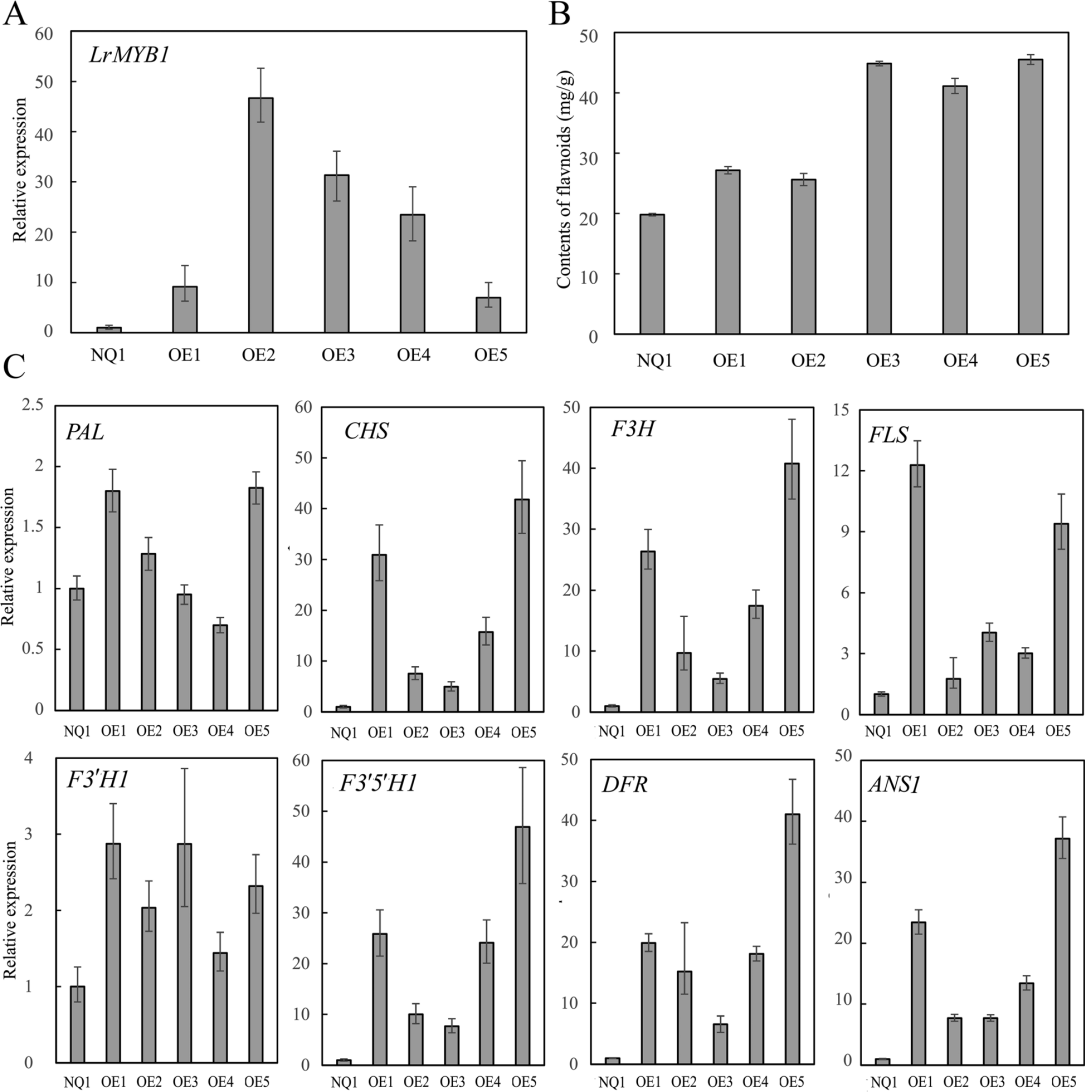
Characteristics of *35S:LrMYB1* overexpression transgenic *L. barbarum* lines. **a** Relative expression of the *LrMYB1* gene; **b** total flavonoids; **c** qRT-PCR analysis of flavonoid biosynthesis genes. NO1, wild type plants of *L. barbarum* ‘Ningqi1’; OE1-OE5, different transgenic lines

To determine the putative function of *LrMYB1* in plants, we generated transgenic *LrMYB1 L. barbarum* plants in which *LrMYB1* was driven by the cauliflower mosaic virus 35S promoter. As a result, the expression of *LrMYB1* was increased more than ten-fold in six independent transgenic lines, suggesting that overexpression was efficient (Fig. 8)a. The seedlings of the T1 generation of transgenic *L. barbarum* lines showed no visible differences in growth in comparison to wild-type lines. We then determined the content of flavonoids in the seedlings and found that the content of total flavonoids was higher in the *35S:LrMYB1* transgenic lines than the wild type (Fig. 8)b.

Several MYB proteins are known to play important roles in the transcriptional regulation of flavonoid biosynthesis genes. To investigate the reason for the increase in flavonoid content, the effect of *LrMYB1* overexpression on eight genes encoding enzymes related to general flavonoid metabolism was examined by qRT-PCR analysis of transgenic seedlings. Interestingly, the qRT-PCR analysis of six independent lines indicated that *LrMYB1* could act as an activator of the expression of different flavonoid structural genes in *Lycium* seedlings. The expression of *PAL* was unaffected by *LrMYB1* overexpression. Expression of the genes encoding CHS, F3H, FLS, F3’H1, F3’5’H1, DFR, and ANS1 was significantly induced in seedlings of transgenic lines in compared with to nontransformed wild-type seedlings (Fig. 8)c.

## Discussion

Both *L. ruthenicum* and *L. barbarum* have been used as traditional medicine in China for several centuries. However, unlike the red ripe fruit of *L. barbarum*, the ripe fruit of *L. ruthenicum* are deep purple in color and have high content of petunidin, which is produced via anthocyanins [2]. The ripe fruit of *L. barbarum* taste sweeter than those of *L. ruthenicum.* In this study, RNA-seq technology was used to determine the key metabolic pathways during ripening of *Lycium* fruit and to unravel the molecular regulatory basis for the differences between two contrasting *Lycium* genotypes. Between the two types of *Lycium* fruit, totals 20,335, 24,469 and 21,056 DEGs in the S1, S2 and S3 stages were identified, respectively. These DEGs were extensively enriched in various pathways, such as phenylalanine metabolism, flavonoid biosynthesis, fructose and mannose metabolism, glycolysis/gluconeogenesis and the pentose phosphate pathway, and might have unique functions in the two cultivated wolfberry genotypes during fruit ripening.

Flavonoids, which are a group of plant polyphenolic secondary metabolites that perform a wide range of physiological functions, are benzo-*γ*-pyrone derivatives consisting of phenolic and pyran rings and are classified according to their substitutions [23–27]. The flavonoid pathway is derived from the general phenylpropanoid pathway, and PAL, a key and rate-limiting enzyme, catalyzes the first step in phenylpropanoid metabolism [28]. In this study, large differences in the transcript level of the *PAL* gene were observed between BF and RF, and the expression of *PAL* was significantly higher in the BF than in the RF (Fig. 4). The active phenylpropanoid pathway might provide more primary materials for other pathways of secondary metabolism in BF than in RF. The flavonoid pathway splits into anthocyanin, proanthocyanidin and flavonol branches after the initial steps. Anthocyanins are responsible for the bright orange, pink, red, violet and blue colors of flowers and fruit of some plant species and play a vital role in human health benefits through their antioxidant activity [29, 30]. The fruit of the two species in the present study are biochemically different in the terms of their accumulation of anthocyanins [16, 31]. Thus, the regulation and activity of the anthocyanin pathway is of interest for improving the nutritional quality of *Lycium*. The anthocyanin pathway has three branches, producing brick-red pelargonidin, red cyanidin, and blue delphinidin pigments. The proportions of the three pathway branches of the anthocyanin metabolism pathway will affect the type and color of anthocyanins in plants [32]. F3’H and F3’5’H are key enzymes responsible for directing the metabolic flux toward the cyanidin and delphinidin branches, respectively [33]. Petunidins, derived from delphinidin, account for approximately 95% of the total anthocyanins in *L. ruthenicum* fruit, while cyanidin and pelargonidin derivatives are not detected [2]. It has also been documented that the ratio of F3’5’H/F3’H transcription controls the composition and proportion of flavonoids in different tissues and different cultivars [16]. In grapes, violet/blue cultivars have a higher ratio of F3’5’H/F3’H transcription than do red cultivars [26]. In this study, the increasing ratio of F3’5’H/F3’H might have partially shifted the metabolic pathway towaed the delphinidin branch in the BF compared with the RF in ripening fruit*.* The expression levels of *PAL*, *CHS*, *CHI*, *F3H*, F3*’H*, F3*’5’H1*, *F3’5’H2*, *DFR1*, *ANS1*, *ANS2*, *CCMT1* and *ANMT* were much higher in the BF than in the RF, and there was hardly any expression in the RF, which might be partly a result of the higher level of anthocyanidins in the RF compared with the BF. The common increase in transcripts may suggest the existence of a common transcription factor that activates the transcription of structural genes for anthocyanin synthesis in BF but does not function in RF. In the well-known anthocyanin pathway in other species, anthocyanin biosynthesis is also regulated by MYB-bHLH-WD40 protein complexes [34, 35]. In this study, *bHLH* presented much higher expression in the BF than in the RF, and its expression increased in the BF but decreased in the RF during fruit development and ripening. These results suggested that bHLH might be candidate transcription factors that controls the anthocyanin pathway.

Sugars are important components in wolfberry fruit and serve as important signals in the regulation of fruit ripening [31, 36]. The in vivo sugar composition in different plant species varies, not only in quantity but also in type. Sucrose, fructose and glucose are the three major components that contribute to the total sugar content in ripe fruit in wolfberry [31]. *L. barbarum* polysaccharide (LBP), a glycoprotein complex, is one of the most important active components in wolfberry, and it has been shown to have many health benefits, including antioxidant, immunomodulation and antitumor activities [37]. In this study, the main sugars in BF and RF were fructose and glucose, whose contents were many times greater than the content of sucrose. The glucose and fructose contents increased throughout fruit development stages both in BF and RF. The contents of glucose and fructose were higher in the RF than in the BF, making the RF taste sweeter than the BF. Additionally, the change trends of the LBP and sucrose contents were surprisingly consistent, implying that sucrose might play a critical role in LBP accumulation, potentially due to its ability to act as a donor of glucosyl or fructosyl residues and due to its roles as an effective precursor molecule for the synthesis of polysaccharides, which has been shown in other species [38]. Sucrose contents were higher in the early stages of fruit ripening but lower in the mature fruit. Consistent with our results, Zheng et al. (2010) and Zhao et al. (2015) reported that the contents of fructose and glucose in *L. barbarum* fruit increased with fruit development [18, 31], and Zheng et al. (2010) also reported that the contents of sucrose first increased and then decreased with fruit ripening [31]. Additionally, many differentially expressed genes were significantly enriched in pathways related to the biosynthesis/catabolism of sugars, such as fructose and mannose metabolism, glycolysis/gluconeogenesis, the pentose phosphate pathway, and starch and sucrose metabolism. Several important genes encoding key sugar metabolism enzymes involved in these complex metabolic pathways were differentially expressed between the RF and BF. In addition, these genes were also differentially expressed during different ripening phases in the BF and RF. Sucrose metabolism lies at the heart of a sensitive, self-regulatory developmental system in plants [17]. Sucrose metabolic enzymes include acid invertase (AI), sucrose synthase (SS) and sucrose phosphate synthase (SPS). The relationship between sugar accumulation and sucrose metabolism-related enzymes has been reported in orange, tomato, and melon, among other species [11, 13, 39–41]. SS is a focal enzyme that can catalyze both the formation and hydrolysis of sucrose in plants, and a positive correlation between SS activity and fruit sucrose accumulation has been observed in watermelon [40]. In this study, the gene expression of one SS homologous gene (SS1) was found to be upregulated in the early stage but then downregulated in the later stage with fruit ripening in RF, while it was upregulated in the later stage of fruit development in BF*.* These changes were noticeably positively correlated with the sucrose content, which suggests that SS1 plays a vital role in sucrose accumulation in *Lycium*. Most plant species contain two nonallelic *SS* genes that exhibit different spatial and temporal expression and are differentially regulated at transcriptional and translational levels [42, 43]. In this study, two genes encoding SS were detected, and SS1 increased during fruit development, while SS2 decreased, suggesting that SS1 and SS2 might play different roles during fruit ripening. SPS is a pivotal enzyme that catalyzes sucrose biosynthesis, and SPS activity positively contributes to the control of flux into sucrose, resulting in sucrose accumulation [40, 41, 44]. In this research, the expression of SPS1, an SPS homologous gene, was consistent with the sucrose content during fruit development in both *Lycium* species, suggesting it plays an important role in sucrose accumulation in *Lycium*. However, SPS2, another SPS homologous gene, showed differenti expression trends during fruit ripening, suggesting its different role in sucrose accumulation. Invertase (AI) irreversibly hydrolyzes sucrose into glucose and fructose [17]. A noteworthy positive correlation between AI and fruit sucrose accumulation has been reported in watermelon [40], melon [45], and tomato [46]. As in other plant species, the expression of AI was consistent with the sucrose content in BF during fruit ripening, but the AI expression was negatively correlated with sucrose accumulation in RF*,* suggesting different mechanisms for sucrose are at play in these two contrasting wolfberry genotypes.

However, to date, little is known about how MYB transcription factors and structural genes coordinate and regulate complex plant growth and development processes, especially in *Lycium*. In our previous studies, 82 MYB proteins were obtained by annotating the transcriptomic data of BF, and their evolution and structural classification were analyzed [19]. To explore the possible MYBs involved in fruit development in BF, we obtained 15 genes with increasing expression and 25 genes with decreasing expression during fruit development. *LrMYB1*, one of the highest expressed differentially expressed *MYB* genes during fruit development, is an ortholog of the *Arabidopsis AtMYB113* gene. In this study, *LrMYB1* was isolated from BF. In *Arabidopsis*, AtMYB113 is known to be involved in flavonoid biosynthesis [47]. MYB transcription factors can be classified into four types (1R-, R2R3-, 3R- and 4R-MYB proteins) based on the number of imperfect repeats (one, two, three or four) in the DNA-binding domain [34]. Our study revealed that LrMYB1 belonged to the R2R3 MYB protein family. Heterogeneous and homogeneous overexpression of MYB genes can induce ectopic flavonoid accumulation in plants. In trees in the genus *Populus*, overexpression of a negative regulator MYB182 in hairy root cultures and whole poplar plants led to reduced proanthocyanin and anthocyanin levels and a reduction in the expression of key flavonoid genes [48]. *PyMYB10*, isolated from Asian pear (*Pyrus pyrifolia*), was sufficient to induce anthocyanin accumulation [49]. In the present study, the *LrMYB1* gene from BF was overexpressed in another *Lycium* species, *L. barbarum*. In the overexpression transgenic lines, the flavonoid content was higher than that in the wild type, suggesting that LrMYB1 regulates flavonoid biosynthesis in *Lycium*. The transcription of genes in the flavonoid biosynthesis pathway investigated by qRT-PCR showed that the expression of a set of genes whose function occurred earlier than those of CHS but not PAL was not regulated. These results suggested that increased flavonoid accumulation resulted from the activation of genes encoding enzymes catalyzing reactions in the upper part of the flavonoid biosynthesis pathway by reducing mRNA levels of genes encoding enzymes involved in flavonoid biosynthesis. Other TFs related to flavonoid biosynthesis, such as other MYBs and bHLHs and WD40s that form complexes with MYB proteins, are still unknown in *Lycium*. Thus, their contribution to flavonoid biosynthesis remains to be determined.

## Conclusion

Since the full-length genome of *Lycium* has not been sequenced, a comparative transcriptome analysis of two contrasting wolfberry genotypes during fruit development and ripening was peformed to provide additional information regarding the genetic basis of variation in fruit development. A significant number of important metabolic pathways and functions associated with unigenes were identified. The sugar contents suggested that fructose and glucose were the most abundant sugars, and that sucrose was important for LBP biosynthesis in *Lycium*. A large number of MYB transcription factors that participate in fruit development and ripening and that could be used for further gene functional analysis were also identified. Moreover, a specific transcription factor, *LrMYB1*, was characterized, and its function in flavonoid synthesis was verified. Hence, our study provides a method for the genetic transformation of *L. barbarum*. In summary, our comparative study provides new genome-wide insights into the molecular-level mechanisms of the fruit quality attributes of wolfberry, such as sugar accumulation and flavonoid synthesis.

## Methods

### Plant cultivation

The tested wolfberry plants were grown at the Goji Germplasm Bank at the Ningxia Forestry Institute (Yinchuan, Ningxia, China; 106°08′~ 107°22′E, 38°28′~ 38°42′N). The elevation of the cultivation site is 1115 m above sea level. It has a temperate continental climate with an annual average temperature of 10.1 °C, an average maximum of 37.2 °C in July, and an average minimum temperature of − 27.9 °C in January. The average annual precipitation is only 181.2 mm, but it is concentrated during the 5 months of the growing season. The mean annual evaporation is 1882.5 mm, and the annual mean sunshine duration is 2800–3000 h.

Two five-year-old samples of wolfberry species, *L. barbarum* ‘Zhongke1’ and *L. ruthenicum*, were selected for transcriptome and molecular metabolic analysis in this study. The colors of the fruit of *L. barbarum* and *L. ruthenicum* are red and black, respectively, and are referred to as red fruit (RF) and black fruit (BF) in this work. First, the flowers of *Lycium* were randomly marked with red wool as soon as they blossomed, and then the phenotype of the fruit epidermis was recorded and evaluated at different times. According to previous evaluations, S1 refers to the green fruit stage at 10 days after flowering, S2 refers to light purple fruit in *L. ruthenicum* and light yellow fruit in *L. barbarum* at 25 days after flowering, and S3 refers to the final stage of ripened fruit that have fully expanded, involving mature black fruit for *L. ruthenicum* and mature red fruit for *L. barbarum*, which occurs 40 days after flowering. Samples of the four fruit developmental stages (for both RF and BF) were harvested based on the phenotype of the fruit epidermis. The fruit samples were harvested in June 2016. There were three biological replicate samples at each developmental stage, and each sample was collected from three different trees. Fresh samples for RNA extraction were randomly collected from both species at the three different ripening stages. These samples were immediately frozen in liquid nitrogen and stored at − 80 °C until use.

### RNA-seq library preparation and sequencing

Total RNA was isolated using the RNA Plant Plus Reagent Kit (TIANGEN, Beijing, China) following the manufacturer’s instructions. The quality, quantity and integrity of total RNA was assessed using a Nano Photometer® spectrophotometer (IMPLEN, CA, USA), a Qubit® RNA Assay Kit in Qubit® 2.0 Fluorometer (Life Technologies, CA, USA) and the RNA Nano 6000 Assay Kit supplied with the Agilent Bioanalyzer 2100 system (Agilent Technologies, CA, USA), respectively. Briefly, 1.5 μg of RNA per sample was used as input material for the RNA sample preparations. First, mRNA was purified from total RNA using poly-T oligo-attached magnetic beads. First strand cDNA was synthesized using a random hexamer primer and M-MLV Reverse Transcriptase (RNase H^−^). Second strand cDNA synthesis was subsequently generated using DNA polymerase I and RNase H. After cDNA library construction, clusters with satisfactory quality were generated according to the manufacturer’s recommendations. The library preparations were sequenced on an Illumina HiSeq™ 2500 at the Novogene Bioinformatics Institute in Tianjin, China.

### Transcript assembly and analysis

High-quality reads (clean reads) were obtained using an eliminated adapter, eliminating reads containing more than 10% unknown nucleotides and low-quality reads (reads containing more than 50% bases with Q-value ≤20). The clean reads were then assembled into contigs using Trinity [50] with min_kmer_cov set to 2 by default and all other parameters set to default. All unigene sequences were aligned by BLASTx to protein databases such as Nr (E-value =1e-5), Pfam (E-value =1e-2), KOG (E-value =1e-3), SwissProt (E-value =1e-5), KEGG (E-value =1e-10), and Nt using BLASTn (E-value =1e-5) databases. The ESTScan tool was used to decide the sequence annotation when a unigene was not aligned to any of the above databases [51]. For NR and Pfam annotation, the Blast2GO program was used to obtain the unigene Gene Ontology (GO) annotation [52]. The quantitation of gene expression is shown in Additional file 9. The repeatability between three biological replicates of each sample was determined based on Pearson Correlation computed by SPSS 22.0, and the results are shown in Additional file 10.

### Functional analysis of DEGs

Differential expression analysis of two groups was performed using the DESeq R package (1.10.1). DESeq provides statistical routines for determining differential expression in digital gene expression data using a model based on the negative binomial distribution. The resulting *P*-values were adjusted using Benjamini and Hochberg’s approach for controlling the false discovery rate [53]. Genes with an adjusted *P*-value < 0.05 found by DESeq were assigned as differentially expressed (FDR < 0.05). Gene expression was calculated with well-mapped reads, and the results were normalized to Fragments Per Kilobase of transcript sequence per Million base pairs sequenced (FPKM). For each unigene, the threshold of FPKM> 0.3 was considered an expressed gene, and the results could be directly used to compare the differences in gene expression between the two groups. DEGs were subsequently carried into GO functional analysis and KEGG pathway analysis.

### GO enrichment analysis

GO enrichment analysis of the differentially expressed genes (DEGs) was implemented using the GOseq R packages based on a Wallenius noncentral hypergeometric distribution [54], which can adjust for gene length bias in DEGs.

### KEGG pathway enrichment analysis

KEGG provides a reference knowledge base for linking genomes to life through the process of PATHWAY mapping, which is to map, for example, the genomic or transcriptomic content of genes to KEGG reference pathways to infer systemic behaviors of the cell or organism [55]. We used KOBAS software to test the statistical enrichment of differential expression genes in KEGG pathways [56].

### Gene expression analysis by qRT-PCR

For each sample, 8 μg of RNA was reverse-transcribed for first-strand cDNA synthesis using the PrimeScript™ RT Reagent kit (TaKaRa, Dalian, China) according to the manufacturer’s protocol. Twenty-four genes that are important for bioactive synthesis were selected to validate the RNA-seq results by qRT-PCR. The primers for qRT-PCR were designed with Primer premier (v5.0) software and are provided in Additional file 11. The actin gene of *Lycium* was used as the internal house-keeping gene control. The 20 μL reaction consisted of 10 μL of SYBR Green Master mix (TaKaRa, Dalian, China), 1 μL of each primer pair and 1 μL cDNA templates. The PCR was performed in 96-well optical reaction plates on a LightCycler® 480 II (Roche, Basel, Switzerland). A total of three biological and three technical replicates were used for each cultivar, and the ripening stage was assayed. The thermal cycling program was as follows: 95 °C for 10 s; 40 cycles of 95 °C for 15 s, and 55 C for 30 s. The results were normalized to the expression level of the constitutively expressed actin gene [57]. The relative quantitative method (2-^△△CT^) was used to evaluate the quantitative variation [58].

### Gene cloning, plasmid construction, and subcellular localization analysis

Based on the partial sequence of the *LrMYB1* gene from the transcriptome analysis, gene specific primers (5’GSP1-F: 5′-CTAATACGACTCACTATAAGGGCAAGCAGTGGATCAAGGC-3′; 5’GSP1-R: 5′-GATTACGCCAAGCTTGGAACTCGATGCCACTTTCCTTCTCC-3′; 5’GSP2-F: 5′-CTAATACGACTCACTATAGGGC-3′; 5’GSP2-R: 5′-GATTACGCCAAGCTTGATTGCTTTTGGTAGATCCTCCCACTG-3′; 3’GSP1-F: 5′-GCGCCTAGGAGGTGATCACTAAAGTGATATCCTTTTTTTTTTTTTTTT-3′; 3’GSP1-R: 5′-GATTACGCCAAGCTTGAACATCTTCTTCTTGAGTCCATGCACC-3′; 3’GSP2-F: 5′-GGAGGTGATCACTAAAGTGATATCC-3′; 3’GSP2-R: 5′-GATTACGCCAAGCTTGTCTGACGTGGTCACCCGATTACCATC-3′) were designed to amplify 5′ and 3′ termini of *L. ruthenicum LrMYB1* using a SMRTer™ RACE cDNA Application Kit (Clontech, Mountain View, CA, USA) according to the manufacturer’s instructions. Then, the obtained 5′ -terminal and 3′ -terminal sequences were used to obtain the full *LrMYB1* cDNA sequence. The specific primers (LrMYB1T-F: 5′-GAATTGATGGTAATCGGGTGAC-3′; LrMYB1T-R: 5′-GTTCAAGCCAATAACGATTGG-3′) were designed based on 5-terminal and 3-terminal sequence information to amplify the full cDNA of *LrMYB1* using high-fidelity PrimeSTAR® Max DNA Polymerase (Takara, Dalian, China) according to the manufacturer’s instructions. Specific PCR products were cloned using a pGEM-T Vector System (Promega, Madison, WI, USA).

The Gateway cloning system (Invitrogen, Carlsbad, CA, USA) was used to construct LrMYB1-GFP fusion and *LrMYB1* overexpression plasmids. The full-length coding sequence (CDS) without the stop codon of the *LrMYB1* gene was amplified by PCR using gene-specific primers with the addition of four extra bases (CACC) at the 5′ end of the forward primer. PCR products were first cloned into entry vector pENTR™/D-TOPO before being subcloned into the destination vector pMDC85 and vector pMDC32, resulting in an in-frame fusion with GFP and a plasmid for overexpression analysis. The cloned construct was introduced into *Agrobacterium tumefaciens* for the following functional analysis in plants.

Young leaves of *Nicotiana tabacum* were used to perform a transient expression assay mediated by *A. tumefaciens* C58 carrying LrMYB1-GFP fusion proteins. The *N. tabacum* plants infiltrated with *A. tumefaciens* C58 were allowed to grow at 23 °C in the dark for 24 h, followed by another 24 h under a 16 h-light: 8 h-dark photoperiod before the leaves were detached for observation of the GFP signal using confocal microscopy.

### Genetic transformation of *L. barbarum*

#### Selection of antibiotics and their concentrations during gene transformation

The bud induction rate of *L. barbarum* ‘Ningqi 1’ from cultured explants was measured, and it was influenced by the concentration of carbenicillin and hygromycin. Therefore, it was measured by adding different concentrations of carbenicillin and hygromycin after the explants had grown for 10 days. When the concentration of carbenicillin was less than 500 mg/L, the rate of bud induction was relatively high, but it was accompanied by a higher infestation rate. The bud induction rate was greater than 20% when the concentration of carbenicillin was 500 mg/L, but it was 0 when the concentration of hygromycin was 20 mg/L. Hence, concentrations of 500 mg/L carbenicillin and 10 mg/L hygromycin were chosen for the bud resistance screening.

#### Agrobacterium-mediated transformation and growth of plants

*A. tumefaciens* carrying the appropriate plasmid was cultured in YEB liquid media supplemented with 10 mg/L rifampicin and 50 mg/L kanamycin, and grown overnight at 28 °C. *A. tumefaciens* was then resuspended in YEB liquid media and grown to an OD_600_ between 0.6 and 0.8 followed by dilution with liquid MS media to an OD_600_ between 0.3 and 0.5.

Transformation of *L. barbarum* ‘Ningqi 1’ by *A. tumefaciens*: 1) aseptic seedling leaves of *L. barbarum* were cut into square blocks of 0.5 cm^2^ (leaf explants) and immersed in Agrobacterium solution for 8 min. The leaf explants were then rinsed several times with sterile water, with excess *A. tumefaciens* suspension removed with sterile filter paper and inoculated into MS media for cocultivation in the dark for 48 h. 2) The leaf explants were washed five times with sterile water and then transferred to resistant callus selection media (0.2 mg/L IAA, 0.5 mg/L 6-BA, 10 mg/L hygromycin, and 500 mg/L carbenicillin for elimination of Agrobacterium, in MS media) for cultivation for approximately 2 months at 27 °C under a 12-h light:12-h dark photoperiod and a light intensity of 2000–2500 lx. The leaf explants were subcultured at least every 7 days to eliminate dead, browned and infested calli. 3) The screened resistant calli were then transferred into fresh resistant bud differentiation media (0.2 mg/L IBA, 0.4 mg/L 6-BA, 10 mg/L hygromycin, and 500 mg/L carbenicillin for elimination of Agrobacterium, in MS media) to induce resistant buds for about 25 days. 4) The resistant buds were transferred to bud proliferation medium (0.4 mg/L IAA, 0.2 mg/L 6-BA, 10 mg/L hygromycin, and 500 mg/L carbenicillin used for elimination of Agrobacterium, in MS media) and differentiation proceeded for approximately 20 days. 5) Last, healthy seedlings that were approximately 2–3 cm in length, were transplanted into rooting media (0.2 mg/L IBA, 500 mg/L carbenicillin in 1/2-strength MS media) to induce rooting for approximately 10 days.

#### Confirmation of transformation

Two or three young leaves of the candidate positive transformed plant shoots and wild-type control shoots were used for plant DNA analysis. A Plant Genomic DNA Extraction Kit (Tiangen, Beijing, China) was employed for DNA extraction according to the manufacturer’s instructions. PCR was performed using candidate transformation plantlets, negative controls (untransformed plantlets) and positive controls (positive plasmids) in a reaction mixture containing approximately 10 ng of template (genomic DNA or cDNA), 1 × PCR buffer, 0.25 mmol/L deoxynucleotide triphosphates, 0.25 mmol/L forward and reverse primer (primer sequence: 5′-GACGCACAATCCCACTATCC-3′ and 5′-CTTCATCAAAGCTCCCAAATG-3′), and 0.5 U of Taq DNA polymerase (Qiagen, Valencia, CA, USA) in a final volume of 20 μL. PCR amplification was performed with an initial denaturation at 94 °C for 4 min, followed by 30 cycles of denaturtion at 94 °C for 30 s, annealing at 55 °C for 30 s, and extension at 72 °C for 60 s; the final extension was performed at 72 °C for 10 min. The amplification products were analyzed by electrophoresis in 0.8% agarose gels and detected by staining with ethidium bromide.

#### Assessment of sugar concentrations

High-performance liquid chromatography (HPLC) was used to optimize the quantitative determination of free sugars in the wolfberry fruit after the frozen fruit were rapidly crushed to a powder in liquid nitrogen using a mortar. Samples (0.5~10 g) were dissolved in 50 mL of water to which 5 mL of both zinc acetate solution (21.9 g/100 mL) and potassium ferrocyanide solution (10.6 g/100 mL) were slowly added, after which the mixture was brought to a final volume of 100 mL with water. Sonication, vibration, centrifugation and suction filter processing were subsequently conducted before HPLC analysis. The optimal HPLC conditions consisted of a mobile phase with a mixture of acetonitrile and water (70:30, v/v) at a flow rate of 1.0 mL/min. The injected sample volume was 20 μL, and the column temperature was 40 °C. Sugar concentrations were determined according to a calibration curve based on a sugar standard solution.

#### Assessment of flavonoid concentrations

The total content of flavonoids in wild-type and *LrMYB1* overexpressing wolfberry seedlings was measured by spectrophotometry using rutin as the standard according to Zhu et al. (2000), with modifications [59]. A vacuum freeze-dryer was used to dry the wolfberry seedlings at − 50 °C, and the freeze-dried seedlings were cushed to a powder with a mortar and stored in a cool and dry place away from sunlight. Afterward, 1 g of powder was weighed, extracted with anhydrous alcohol and petroleum ether (99.8:0.2, V/V) sonicated and centrifuged, after which the volume was brought to 50 mL with alcohol solution (60%) before analysis. The appropriate volume of the solution was then taken and dissolved in 50 mL of water, to which 0.8 mL of aluminum nitrate solution (50 g/L), 0.8 mL of potassium acetate solution (100 g/L) and 10 mL of sodium hydroxide solution (40 g/L) were slowly added, after which the final volume was then brought to 100 mL with an alcohol solution (60%). The absorbance was measured at 510 nm. The flavonoid concentration was assessed using a calibration curve generated with the rutin solution.

## Supplementary information

**Additional file 1 **Characteristics of assembled unigenes. The figure showing the length distribution of unigene in two *Lycium* species.

**Additional file 2 **DEGs in two *Lycium* species at three developmental stages. The file showing differentially expressed genes between BF and RF at three developmental stages.

**Additional file 3.** GO enrichment analysis of DEGs at three developmental stages.

**Additional file 4.** KEGG pathway annotations of DEGs at three developmental stages.

**Additional file 5 **KEGG pathway enrichment analysis of DEGs of RF and BF during fruit development and ripening. BF: Black fruit of *L. ruthenicum*; RF: red fruit of *L. barbarum*; (A) 10 DAF; (B) 25 DAF; (C) 40 DAF.

**Additional file 6 **FPKM values of the anthocyanin- and sugar-related genes in the fruit of the two *Lycium* species. The figure showing FPKM values of the selected anthocyanin- and sugar-related genes in the fruit of the two *Lycium* species.

**Additional file 7 **FPKM values of the anthocyanin- and sugar-related genes of each biological replication in the fruit of the two *Lycium* species. S1: 10 DAF; S2: 25 DAF; S3: 40 DAF.

**Additional file 8 **Sequences and typing information for 82 MYB transcription factors identified from transcriptome sequencing in *L. ruthenicum*.

**Additional file 9.** Quantitation of gene expression. File showing the read counts and FPKM values of the genes in each sample. (XLS 18854 kb)

**Additional file 10.** Pearson correlations between each pair of samples based on FPKMs. A: RF; B: BF. The repeatability between three biological replicates of each sample was determined based on Pearson Correlation computed by SPSS 22.0.

**Additional file 11.** Primer sequence information used for qRT-PCR-based validation of the RNA-seq data.

## Data Availability

The datasets supporting the results presented in this manuscript are included within the article (and its additional files). The raw reads for the 18 sequenced libraries are available in the NCBI SRA database under number PRJNA483521 [https://www.ncbi.nlm.nih.gov/sra/?term=PRJNA483521&utm_source=gquery&utm_medium=search].

## Abbreviations

AI: Invertase
ANMT: Anthocyanin methyltransferase
ANR: Anthocyanidin reductase
ANS: Anthocyanin synthase
C3’H: P-coumaroyl shikimate 3′-hydroxylase
C4H: Cinnamic acid 4-hydroxylase
CCMT: Caffeoyl-CoA O-methyltransferase
CHI: Chalcone isomerase
CHS: Chalcone synthase
DFR: Dihydroflavonol 4-reductase
F3’5’H: Flavonoid-3′,5′-hydroxylase
F3’H: Flavonoid 3′-hydroxylase
F3H: Flavanone 3-hydroxylase
FLS: Flavonol synthase
PAL: Phenylalanine ammonia-lyase
SPS: Sucrose phosphate synthase
SS: Sucrose synthase
UGAT: Cyanidin-3-O-glucoside 2-O-glucuronosyltransferase
