# Supplementary figures and images for: Comparative transcriptome analysis of two contrasting wolfberry genotypes during fruit development and ripening and characterization of the LrMYB1 transcription factor that regulates flavonoid biosynthesis
Source: BMC Genomics. 2020 Apr 10;21:295. doi: 10.1186/s12864-020-6663-4 (PMC7147035; doi:10.1186/s12864-020-6663-4)

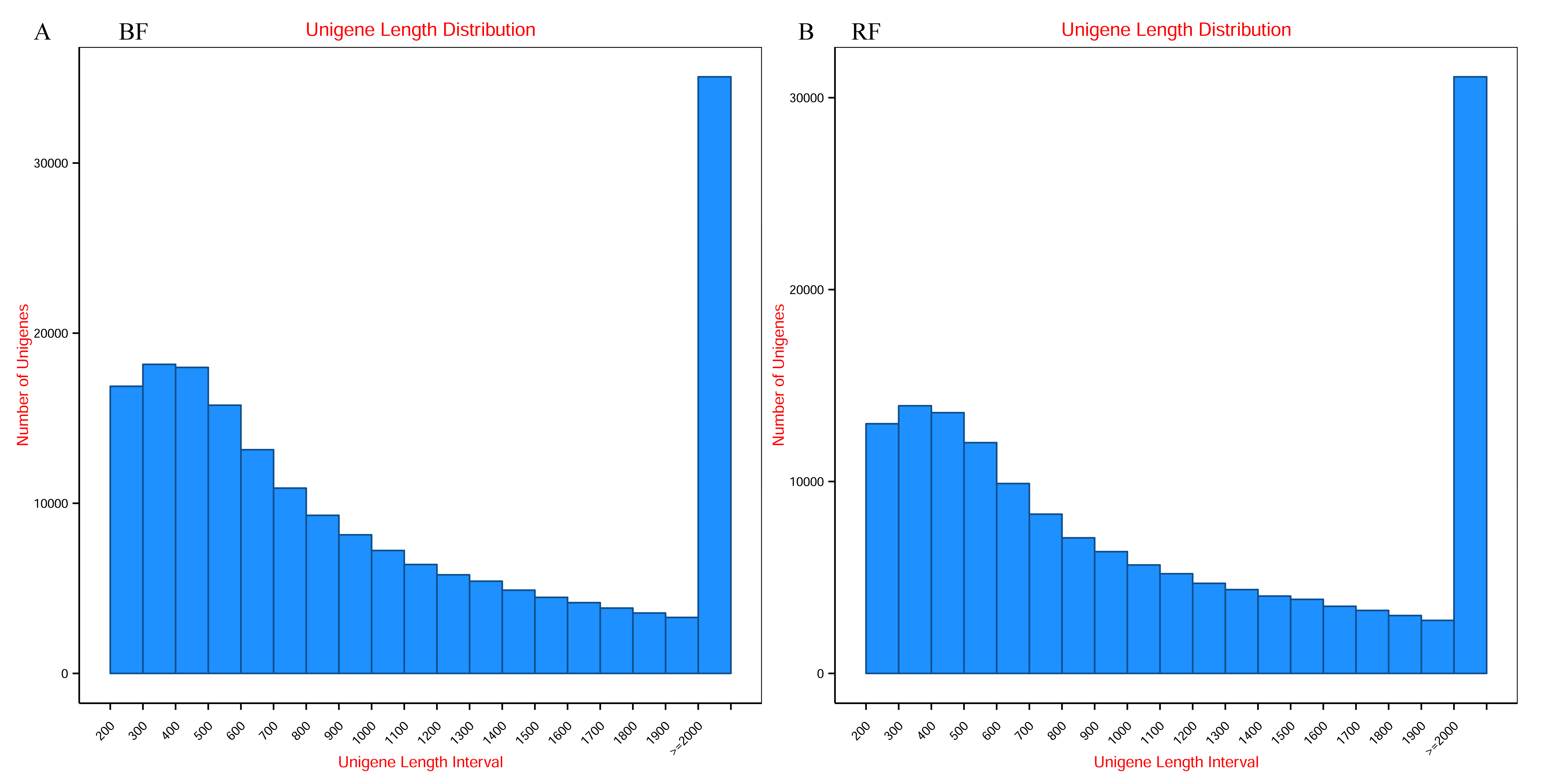

Supplement: Supplementary file 1 — Additional file 1 Characteristics of assembled unigenes. The figure showing the length distribution of unigene in two Lycium species. [file 12864_2020_6663_MOESM1_ESM.jpg]

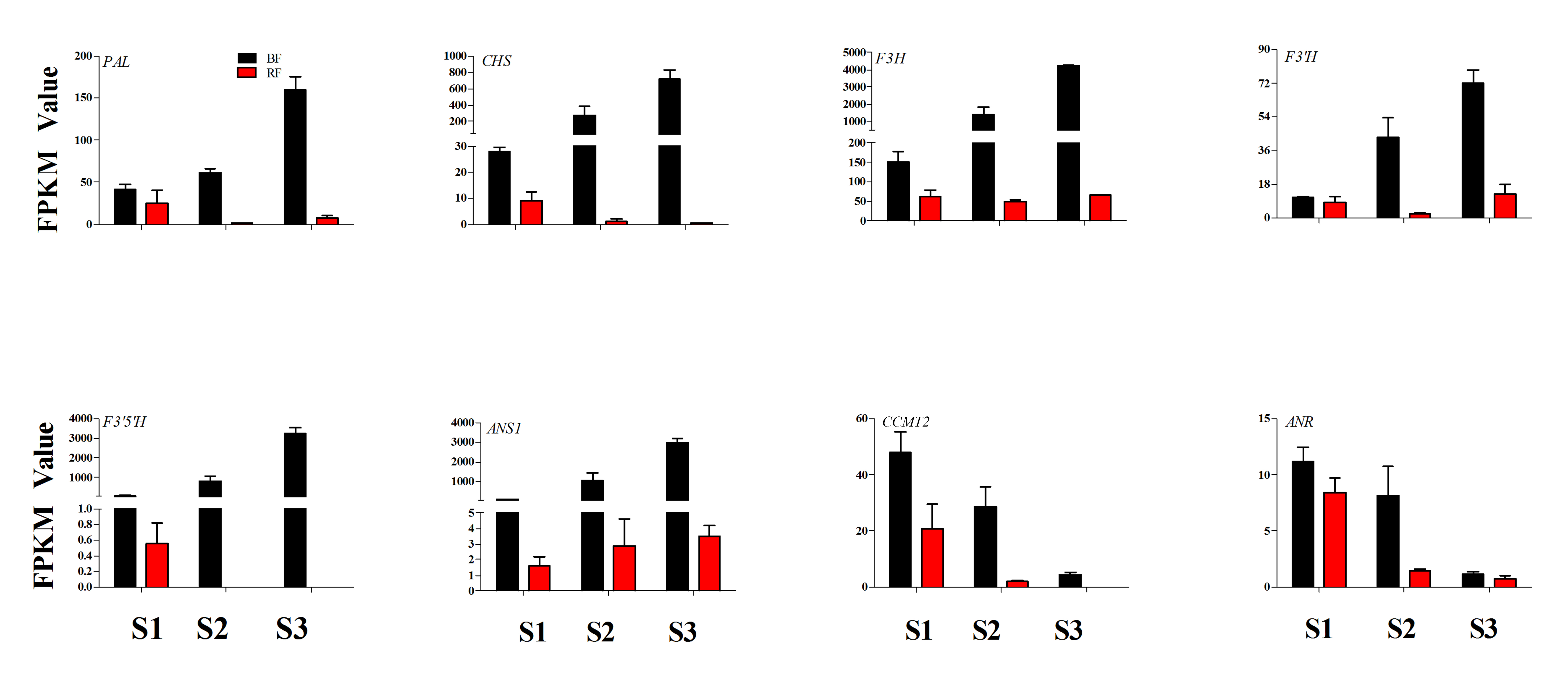

Supplement: Supplementary file 6 — Additional file 6 FPKM values of the anthocyanin- and sugar-related genes in the fruit of the two Lycium species. The figure showing FPKM values of the selected anthocyanin- and sugar-related genes in the fruit of the two Lycium species. [file 12864_2020_6663_MOESM6_ESM.tif]

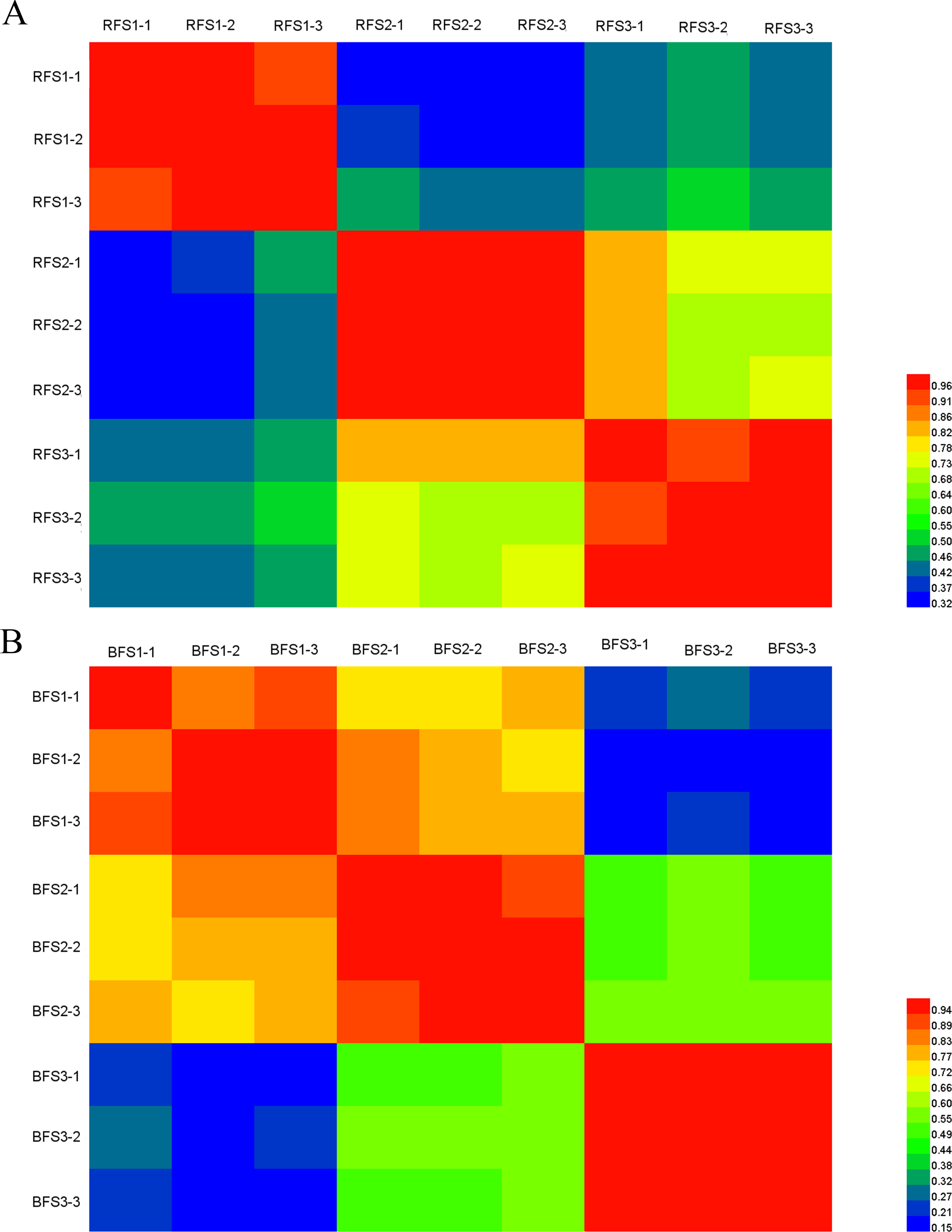

Supplement: Supplementary file 10 — Additional file 10. Pearson correlations between each pair of samples based on FPKMs. A: RF; B: BF. The repeatability between three biological replicates of each sample was determined based on Pearson Correlation computed by SPSS 22.0. [file 12864_2020_6663_MOESM10_ESM.tiff]
