# Supplementary material for: Comparative transcriptome analysis of two contrasting wolfberry genotypes during fruit development and ripening and characterization of the LrMYB1 transcription factor that regulates flavonoid biosynthesis
Source: BMC Genomics. 2020 Apr 10;21:295. doi: 10.1186/s12864-020-6663-4 (PMC7147035; doi:10.1186/s12864-020-6663-4)
Supplement: Supplementary file 11 — Additional file 11. Primer sequence information used for qRT-PCR-based validation of the RNA-seq data. [file 12864_2020_6663_MOESM11_ESM.docx]

Table S1 Primer sequences information used for qRT-PCR validation of RNA-seq data.

| Gene name | Forward primer (5’-3’) | Revese primer (5’-3’) |
| --- | --- | --- |
| PAL | AGGATGAACAGGATGCCAATGC | CATCAAAGGGGATGCTAAGCAGTAC |
| C4H | CGAGTGGGGTATCGCTGAACTAG | AAGAGTCTCCTTGACCACAGCCT |
| C3’H | GAGGAGCTAGACCGGGTTATCG | GAGGGAGCATTAGTGGAGTTGGAG |
| CHS | GGCAATTCTGGACCAAGTTGAAC | ACCAGTGCTACCAAGCCCTTCT |
| CHI | CCTGACACTGGGAATGCTGTTATAG | ACAAGGATTGCTCAATTTCTGG |
| F3H | CAACACTAACGGCTCTAGCGGAT | CAATACCCTTCAAGGATATGACT |
| F3’H | TGCCACTACCACCAGGTCCAA | ACCACGTCCACAAAACCCATC |
| F3’5’H1 | TTCCGAAAACACCCTTCAACAC | CATGAACCTCTCAGGATCGAACT |
| F3’5’H2 | TGGACCTGTTATGTATCTCAAAG | CGTTATAGGCCAAGTGTGTCG |
| DFR1 | CCCAAGGCAGAGGGAAGATAC | CCATGTCCATTAGCTTCTTTGACG |
| DFR2 | GCTGCTATCATCACTCACACAAAGA | TGTCTAAGCACGCAAACAGTCG |
| ANS1 | AGGATGAACAGGATGCCAATGC | CATCAAAGGGGATGCTAAGCAGTAC |
| ANS2 | TCCATTGATTGACCTAGAAAACC | AACTCTCGCCAAACACCACG |
| CCMT1 | CACTGGATACTCTTTGCTCCTCACA | CTAATAATGCTGGCGACTGAATGAA |
| CCMT2 | GAACTCACCAGCTTTACCTGTTC | CCAACTGCACTAGTCTCTCGTG |
| ANMT | GACGATAGAGATTGGAGTTTTCAC | TGGTAATCCAACCTCATATGC |
| UGAT | CAACCAATGAATGCTAGAGTTGTG | CTCTAACTTTCTTCCTCACGCC |
| BHLH | AGATGGCTCGAATAATATGGACTC | TGGTAAATGTTGAAGGTCTTGGAAGT |
| ANR | GGATACCCTGTCTCCAAAACATTAG | GGAGTTTCTGGAGTAAGGTAAGGAC |
| AI | GGCAATGGCTGCTAACC | CACTCCCACATACCCGTAC |
| SS1 | TCTGAGGCCTGGTGTGTGG | GGTTGGTTTGGGAAATGATG |
| SS2 | GAGACTTTCATTGAGGATGTCGC | GATACTTTGTTTTCTCCAACGCATG |
| SPS1 | GTCAAGTCGCTTATCCCAAAC | GCTGCTGCCTCAATCAAAG |
| SPS2 | TCCAATGGTCCTAACAGGAC | CACCTTCAATCCTCCTCATG |
| MYB1 | TTGTGAGAAGCCAACAGGTG | CTTCATCAAAGCTCCCAAATG |
| MYB3 | GATCATAAACAAGGAGGAAATGC | ATCCATCTCAGCCTGCAAC |
| MYB7 | TGATAGCGGAACGACACATC | GACTTTCATTGCCAGTGAGC |
| MYB25 | CTTCAGTTCCTCGTTTGAAATG | CAGGTTTCTGTACATCTGGAGATG |
| MYB30 | CTGCTGCCTTCCCTACTACA | GAGGTTCTTCATCTTTCGGTC |
| MYB34 | GGACGGACGATTTATCCAC | AGTCCCCAGACTTTATGAAAAC |
| MYB44 | GTTCACTTACCTGGAAGATCAGC | GCTGGACTGGTAGCTGTTATTAG |
| MYB73 | CTTCTTCAGAGCCCACATGG | CATGGTGACAGACTACCCGATTC |
| MYB77 | GATCCGCCGACTTCTCTTAG | CCTTGTCTTCCTGCTGTGG |
| MYB86 | GAGACAGGATGGGAGGAAAC | GCAAGCTGGCATTGTTAGTAAC |
| MYB95 | CGAAGCCAZTZZCAACTGATG | GAAATAACCTTTCCACCTTCC |
| AN2 | CTCTTCCTGCATCTATTCAATCC | GTGAGCTGGCCTACATCATG |
| ACTIN | AACCAACTGCTGAACGGGAAAT | CATGGATGGCTGGAAGAGGAC |
